# LncPNdeep: A long non-coding RNA classifier based on large language model with peptide and nucleotide embedding

**DOI:** 10.1016/j.ncrna.2026.06.004

**Published:** 2026-08-13

**Authors:** Zongrui Dai, Feiyang Deng, Hsiao H. Sung

**Affiliations:** a Department of Biostatistics, University of Michigan, 1415 Washington Heights, Ann Arbor, MI, 48109, USA; b Department of Oral and Maxillofacial Surgery, University of Michigan, 1011 N. University, Ave, Ann Arbor, MI, 48109, USA; c Experimental Rheumatology, Department of Rheumatology, Radboud University Medical Centre, Nijmegen, the Netherlands

**Keywords:** Long non-coding RNA, Deep learning, Feature embedding, Masked language model

## Abstract

Accurate classification of long non-coding RNAs (lncRNAs) is essential for transcriptome annotation and understanding gene regulation. Existing computational methods predominantly rely on nucleotide sequence features, frequently overlooking biologically relevant peptide signals encoded within lncRNAs. To overcome this limitation, we developed LncPNdeep, an integrative deep learning framework that combines nucleotide and peptide embeddings extracted via masked language models, specifically utilizing contextual representations from BigBird, Longformer, and ProtTrans. By fusing both features in a concatenated neural architecture, LncPNdeep robustly captures complex sequence relationships and improves discrimination between lncRNAs and coding RNAs. Benchmarking on the human transcriptome achieved state-of-the-art performance with 97.1% accuracy, surpassing established lncRNA classification tools and baseline machine learning models. LncPNdeep also demonstrated superior generalization ability across cross-species datasets, maintaining consistently high accuracy and F1 scores. Permutation analysis highlighted the pivotal role of peptide embeddings, especially Average Peptide Embedding, in model performance, while t-SNE visualizations confirmed that integrating multiple embeddings markedly enhances the separation of lncRNAs from coding RNAs. These results position LncPNdeep as a versatile and powerful tool for transcriptomic research, facilitating lncRNA discovery, biomarker identification, and comparative genomics. The model and instructions are freely available at https://github.com/yatoka233/LncPNdeep.

## 1 Introduction

Long non-coding RNAs (lncRNAs) comprise a heterogeneous class of RNA molecules longer than 200 nucleotides that lack protein-coding potential [1]. Nonetheless, they play pivotal roles in gene regulation and developmental processes, including X chromosome inactivation, genomic imprinting, and Hox gene expression [[2], [3], [4], [5]]. Dysregulated lncRNAs have been implicated in major diseases such as cancer, cardiovascular disorders, and Alzheimer's disease [[6], [7], [8]], highlighting the need for robust computational methods to accurately identify and characterize lncRNAs for biomarker and therapeutic applications.

A variety of classification approaches have been developed, with most relying on nucleotide sequence features analyzed via machine learning or deep learning. For example, CPC2 leverages support vector machines (SVMs) with features derived from random forests [9], while PLEK enhances SVMs with improved k-mer spectra [10]. CPAT evaluates coding potential using logistic regression trained on metrics like the Fickett Score, Hexamer Score, and ORF features [11]. CNCI uses adjacent nucleotide triplet frequencies and dynamic programming for SVM training [12], and FEELnc incorporates random forest models based on k-mer and ORF features [13]. More recent models employ ensemble strategies or deep neural networks to classify lncRNAs [14,15].

Advances in deep learning have enabled the extraction of abstract sequence features. DeepPlnc uses dual convolutional neural networks (CNNs) for parallel analysis of nucleotide and structural information [16], while PlncRNA-HDeep combines CNN and LSTM layers to automate feature learning [17]. Multimodal frameworks such as lncRNA-Mdeep and lncRNA-MFDL integrate features from ORFs, k-mers, and sequence encodings using stacked neural architectures [18,19]. However, the overwhelming focus on nucleotide-derived features in these methods risks missing biologically relevant peptide-encoded signals.

Recent discoveries reveal that some lncRNAs encode short, functional peptides derived from small open reading frames (sORFs) [20]. Recent studies using ribosome profiling, mass spectrometry, and evolutionary analysis demonstrate that a subset of lncRNAs encode short peptides from sORFs that are actively translated, developmentally regulated, and in some cases subject to natural selection, indicating they are not translational noise. These lncRNA-derived peptides are typically shorter, more context-specific, and functionally distinct from canonical proteins, supporting the use of peptide-level information as a complementary signal for distinguishing lncRNAs from protein-coding transcripts [[21], [22], [23]]. These peptides possess unique structural and biological properties distinct from canonical proteins, suggesting that peptide information can improve the classification of lncRNAs versus coding RNAs [[24], [25], [26], [27]]. Incorporating both nucleotide and peptide features therefore presents a promising direction for more accurate lncRNA identification.

Large language models (LLMs), originally developed for natural language processing, are highly effective at capturing complex relationships within sequential data, including biological sequences [28,29]. By leveraging both nucleotide and peptide contexts, LLMs can overcome limitations of handcrafted feature engineering and enhance model performance. LLM-based architectures have shown state-of-the-art results in diverse tasks such as sequence classification and question answering [30,31], while self-supervised and semi-supervised pre-training allow generalized features to be learned from raw data [[32], [33], [34]].

## 2 Materials and methods

### 2.1 Dataset

Coding RNAs and lncRNAs for *Homo sapiens* were obtained from GENCODE Release 43 (GRCh38.p13) [35], initially comprising 54,291 lncRNAs and 110,224 coding RNAs. For this study, the training set included 48,876 lncRNAs and 99,187 coding RNAs, while the test set consisted of 5415 lncRNAs and 11,037 coding RNAs (Table 1). To balance the training data, we performed bootstrapping, resampling lncRNAs so that both classes contained 99,187 samples each.

**Table 1:** The human RNA dataset separation for training and testing.

	LncRNAs	Coding RNAs
Train dataset	48,876	99,187
Test dataset	5415	11,037
Total	54,291	110,224

For cross-species validation, we curated datasets from six vertebrates and six fungi species using the Ensembl and Ensembl Fungi databases [36]. Vertebrate species included panda (*Ailuropoda melanoleuca*), cow (*Bos taurus*), zebrafish (*Danio rerio*), chicken (*Gallus gallus*), gibbon (*Nomascus leucogenys*), and pig (*Sus scrofa*). Fungi species included *Aspergillus nidulans*, *Magnaporthe grisea*, *Puccinia graminis*, *Saccharomyces cerevisiae*, *Schizosaccharomyces pombe*, and *Zymoseptoria tritici*.

### 2.2 Peptide embedding

To incorporate peptide-level information, we extracted embedding using ProtTrans [37], a state-of-the-art protein language model pretrained on billions of protein sequences. Because many lncRNAs do not contain annotated or detectable ORFs, we designed three peptide representations per transcript, Fake Peptide Embedding, Max Peptide Embedding, and Average Peptide Embedding, generated through a unified ORF-scanning and translation pipeline.

Specifically, we first scanned each RNA sequence to identify candidate ORFs by locating start codons (ATG) and stop codons (TAG, TGA, TAA). When no ORF was detected, we constructed a pseudo-peptide by parsing the entire nucleotide sequence into non-overlapping codons in a fixed reading frame starting from the first nucleotide (positions 1–3, 4–6, 7–9, …) and translating each codon using the standard genetic code. We adopted this translation-based fallback (rather than a standard k-mer encoding) to preserve local triplet structure while remaining compatible with protein-language-model inputs. When one or more ORFs were detected, we translated the longest ORF to produce the Max Peptide Embedding, and we translated all detected ORFs and averaged their ProtTrans embeddings to compute the Average Peptide Embedding. For transcripts without detectable ORFs, both the Max and Average embeddings were set equal to the Fake Peptide Embedding, ensuring a consistent peptide input representation across all samples.

These three embeddings capture translation-related information at complementary granularities. The Average Peptide Embedding summarizes patterns across all detected ORFs and provides a broad peptide-level characterization; the Max Peptide Embedding emphasizes strong coding-like signals by focusing on the longest ORF; and the Fake Peptide Embedding provides a minimal but stable peptide representation for ORF-absent transcripts, enabling uniform integration of peptide features within the fused deep learning framework.

### 2.3 Nucleotide embedding

We generated nucleotide embeddings by adapting the Masked Language Model (MLM) framework to RNA sequences, enabling efficient learning of contextual relationships. Each base of the RNA sequence (A, C, G, U) was treated as a discrete token. To facilitate classification, a special token ([CLS]) was added to the beginning of every sequence, and 15% of tokens were randomly replaced with the mask token ([MASK]) during MLM-style pre-training, with the objective of reconstructing the original sequence via prediction at masked positions.

Each token $x_{i}$ was initially transformed to a one-hot vector of length N, representing the number of unique tokens (including [CLS] and [MASK]). The one-hot vector was then projected to a d-dimensional embedding space using a trainable embedding matrix $V_{emb}$:

(1.) $$\begin{array}{c} t_{i}^{emb}=V^{emb}\cdot x_{i}\text{,} \end{array}$$

The transformer architecture then facilitated complex interactions among positions in the sequence through the attention mechanism. The attended representation at position i was computed as:

(2) $$\begin{array}{c} t_{i}=\sum_{j=1}^{length}\alpha_{i,j}W_{value}t_{j}^{emb} \end{array}$$

where the attention scores $\alpha_{i,j}$ between position i and j were calculated by:

(3) $$\begin{array}{c} \alpha_{i,j}=\frac{\exp\left(\frac{1}{\sqrt{d}}\left\langle W_{query}t_{i}^{emb},W_{key}t_{j}^{emb}\right\rangle \right)}{\sum_{j^{\prime }}^{length}\exp\left(\frac{1}{\sqrt{d}}\left\langle W_{query}t_{i}^{emb},W_{key}t_{ij^{\prime }}^{emb}\right\rangle \right)}\text{,} \end{array}$$

Here, $W_{query}$, $W_{key}$, and $W_{value}$ are trainable weight matrices. For richer feature representation, multi-head attention concatenates the outputs from multiple heads.

After attention, residual connections and a feed-forward network (FFN)—typically structured as two linear layers with a non-linear activation—enhanced the stability and non-linearity of the model:

(4) $$\begin{array}{c} t_{i}^{res}=t_{i}^{emb}+W_{res}t_{i}\text{,} \end{array}$$

(5) $$\begin{array}{c} t_{i}^{{layer}_{1}}=t_{i}^{res}+FFN\left(t_{i}^{res}\right)\text{,} \end{array}$$

The input dimension and output dimension for each transformer layer are the same. In practice, multiple layers of transformers are stacked together to get the output embedded

(6) $$\begin{array}{c} t_{i}^{emb}\to t_{i}^{{layer}_{1}}\to \cdots t_{i}^{{layer}_{l}}\text{.} \end{array}$$

Finally, *t*^*layer*^*l* can be followed by another FFN to predict the original token and calculate the cross-entropy loss to train the model.

Many lncRNAs exceed the typical length capacity of standard transformer architectures. The resulting large attention matrices introduce computational challenges for long sequences. To address this, we leveraged BigBird and Longformer, which are specifically designed for efficient modeling of lengthy inputs [[38], [39], [40], [41], [42]]. For each RNA, we sampled k non-overlapping subsequences, each constrained within a length window [l,u], defined by the maximum input capacity for each model.

During prediction, the output embedding corresponding to the [CLS] token from each subsequence served as its summarized sequence representation. To obtain an overall feature for each RNA, we averaged the [CLS] outputs across all sampled subsequences. Thus, three types of nucleotide embeddings were generated for each RNA sequence: Longformer256, BigBird256, and BigBird768, corresponding to model and embedding dimensions.

### 2.4 Concatenated deep learning

After generating peptide (Fake, Max, and Average Peptide) and nucleotide (Longformer256, BigBird768, and BigBird256) embeddings, each embedding was independently processed through a custom CNN model consisting of four 1D convolutional layers (kernel size = 3, filters = 128, with residual connections) to enhance feature extraction and maintain stable gradient flow. The output from all CNNs were concatenated to form a single feature vector of dimension 4,352, which was regularized with a dropout layer (rate = 0.8) to mitigate overfitting.

**Fig. 1:**
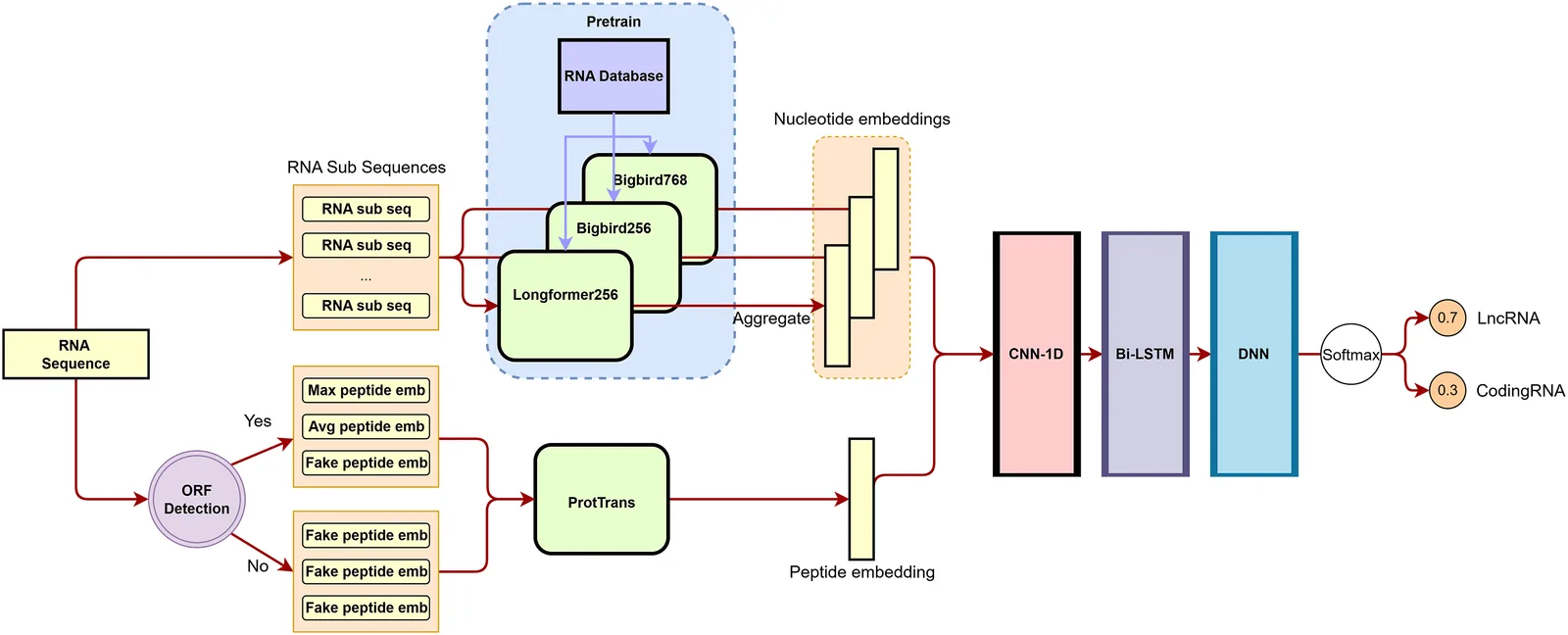
**Overview of the LncPNdeep framework for lncRNA classification.** A deep learning framework integrating peptide and nucleotide embeddings for lncRNA classification. The model processes RNA sequences from a database through parallel embedding pipelines: (Left) Nucleotide embeddings are extracted from RNA subsequences using long-sequence transformer models (BigBird-768 and Longformer-256), followed by aggregation. (Right) Peptide embeddings are generated via ProtTrans from potential open reading frames (ORFs). These embeddings are processed through separate 1D convolutional neural networks (CNNs) with residual connections, concatenated, and passed to bidirectional LSTM (Bi-LSTM) and dense neural network (DNN) layers for final classification into lncRNA or coding RNA categories.

This vector was then fed into two bidirectional LSTM (BiLSTM) layers (256 units each), designed to model sequential dependencies and contextual relationships among the combined features. The outputs were passed to a dense neural network (DNN) layer with softmax activation for binary classification, with categorical cross-entropy used as the loss function. An overview of the full pipeline is shown in Fig. 1.

Together, these components constitute an end-to-end framework that robustly integrates heterogeneous sequence features from both peptide and nucleotide domains. This concatenated deep learning framework provided a unified and robust approach for lncRNA 160 classification.

The model and instructions are freely available on GitHub at https://github.com/yatoka233/LncPNdeep, and the pre-trained model weights have been uploaded and organized on Hugging Face at https://huggingface.co/yatoka/LncPNdeep.

## 3 Results

To evaluate the predictive performance of LncPNdeep and existing tools, we applied our model to both the human transcriptome and cross-species datasets. For human RNA classification, we compared LncPNdeep with six established methods: CNCI, CPC2, CPAT, PLEK, LncAdeep, and LncRNA Mdeep. All models were benchmarked on a unique test dataset; models with retraining capability (PLEK and CPAT) were re-trained using the same training data as LncPNdeep, while pre-trained models were used for CNCI, CPC2, LncAdeep, and LncRNA Mdeep due to technical limitations or lack of retraining options.

### 3.1 Performance of human lncRNA identification

To further validate LncPNdeep's effectiveness, we benchmarked its performance against traditional machine learning algorithms, including Support Vector Machine (SVM), Logistic Regression (LR), and Random Forest (RF). Each baseline model was trained and tested on the same human transcriptome datasets, utilizing one of six available embeddings—three peptide embeddings (Max, Average, and Fake) and three nucleotide embeddings (Longformer256, BigBird256, and BigBird768)—per training instance.

Among baseline models, SVM with BigBird256 embeddings achieved the highest accuracy (Accuracy = 0.93), with BigBird256-based embeddings outperforming the other types (Table 2).

**Table 2:** Performance comparison of traditional machine learning models (Support Vector Machine, Random Forest, and Logistic Regression) trained on different single embeddings, against the pro-posed LncPNdeep model which integrates all embeddings.

Model Embedding	Feature	Accuracy	Precision	Recall
**LncPNdeep**	All	**0.95**	**0.90**	**0.95**

RF	Max Peptide	0.83	0.75	0.73
	Aver Peptide	0.84	0.76	0.74
	Fake Peptide	0.84	0.76	0.74
	Bigbird256	0.92	0.86	0.92
	Bigbird768	0.88	0.80	0.86
	Longformer256	0.86	0.81	0.73
LR	Max Peptide	0.81	0.70	0.73
	Aver Peptide	0.79	0.67	0.73
	Fake Peptide	0.83	0.73	0.75
	Bigbird256	0.92	0.86	0.92
	Bigbird768	0.88	0.79	0.85
	Longformer256	0.85	0.78	0.76
SVM	Max Peptide	0.80	0.67	0.75
	Aver Peptide	0.75	0.62	0.63
	Fake Peptide	0.82	0.70	0.77
	Bigbird256	0.93	0.86	0.94
	Bigbird768	0.87	0.76	0.89
	Longformer256	0.87	0.79	0.81

In direct comparison with the six established lncRNA classification methods, LncPNdeep achieved 97.1% accuracy, 96.7% specificity, and 98.0% sensitivity on the human RNA test dataset (Table 3). Notably, LncPNdeep's sensitivity exceeded all existing methods, with margins of improvement of 3.6%, 7.0%, 27.3%, 17.5%, 64.8%, and 28.4% compared to LncAdeep, LncRNA Mdeep, CPC2, CNCI, CPAT, and PLEK, respectively.

**Table 3:** Performance comparison of LncPNdeep with six existing lncRNA classification methods on the human dataset.

	Accuracy	Specificity	Sensitivity
LncPNdeep	**0.971**	**0.967**	**0.980**
LncAdeep	0.951	0.962	0.944
LncRNA_Mdeep	0.922	0.944	0.910
CPC2	0.781	0.921	0.707
CNCI	0.855	0.951	0.805
CPAT	0.539	0.965	0.332
PLEK	0.771	0.914	0.696

### *3.2* Performance of cross-species lncRNA identification

To assess the generalization capability of LncPNdeep, we evaluated its performance on RNA datasets from 12 different species, using only the human RNA dataset for training. LncPNdeep demonstrated high accuracy and F1 scores across most species, with particularly strong results in panda (*Ailuropoda melanoleuca*, 95.5% accuracy, 97.6% F1), cow (*Bos taurus*, 93.5% accuracy, 97.7% F1), and zebrafish (*Danio rerio*, 90.6% accuracy, 96.3% F1) (Table 4).

**Table 4:** Cross-species performance comparison of LncPNdeep and four other methods. Training was conducted only on human RNA, and testing was performed on six vertebrates and six fungi species. Metrics include accuracy and F1 score.

	LncPNdeep		CNCI		CPAT		CPC2		PLEK	
	Acc	F1	Acc	F1	Acc	F1	Acc	F1	Acc	F1
Pandas	**0.955**	**0.976**	0.900	0.940	0.944	0.970	0.919	0.649	0.785	0.873
Cow	0.935	**0.977**	0.922	0.949	**0.95**	0.972	0.942	0.817	0.825	0.894
Zebrafish	**0.906**	**0.963**	0.877	0.918	0.894	0.940	0.815	0.547	0.683	0.785
Chicken	**0.943**	**0.946**	0.937	0.937	0.924	0.940	0.920	0.895	0.815	0.828
Gibbons	**0.929**	0.941	0.895	0.920	0.913	**0.952**	0.904	0.739	0.736	0.837
Pig	**0.945**	0.910	0.929	**0.932**	/	/	0.923	0.849	0.762	0.808
*A.nidulans*	**0.956**	**0.976**	0.911	0.951	0.950	0.974	0.939	0.413	0.405	0.575
*M.oryzae*	**0.890**	**0.954**	0.785	0.874	0.877	0.934	0.846	0.238	0.534	0.696
*P.graminis*	**0.906**	**0.959**	0.787	0.874	0.865	0.927	0.897	0.379	0.298	0.458
*S.cerevisiae*	**0.890**	**0.950**	0.677	0.804	0.823	0.902	0.107	0.193	0.485	0.650
*S.pombe*	0.872	**0.940**	0.824	0.823	0.843	0.899	**0.941**	0.906	0.697	0.751
*Z.tritici*	0.952	0.964	0.884	0.938	**0.957**	**0.978**	0.878	0.179	0.247	0.395

While LncPNdeep performed robustly, there were a few exceptions where other methods marginally outperformed it in terms of accuracy: CPAT achieved the highest accuracy on cow (95.0%) and *Zymoseptoria tritici* (95.7%), while CPC2 led in *Schizosaccharomyces pombe* (94.1%). Despite these cases, LncPNdeep maintained F1 scores above 90% for all species tested, underscoring its strong robustness and reliability in cross-species lncRNA identification.

Performance degradation in fungal species likely reflects substantial divergence from vertebrate transcriptomes, including differences in codon usage bias, ORF length distributions, transcript compactness, and evolutionary constraints. These factors may reduce the transferability of representations learned from human data, particularly for peptide-level features. In addition, fungal lncRNA annotations are less standardized, which may further affect evaluation consistency.

### 3.3 Permutation analysis for each peptide embedding

**Fig. 2:**
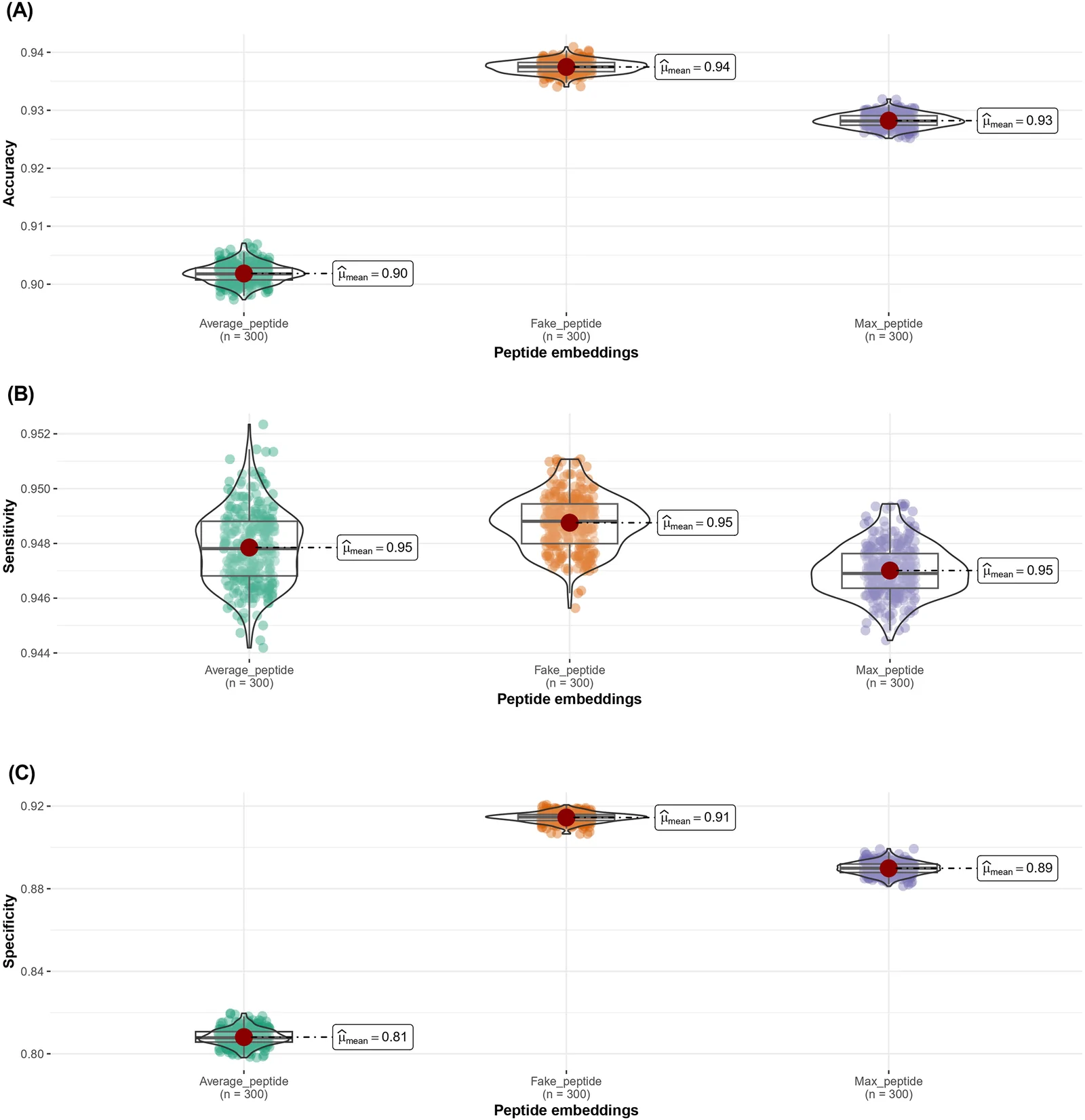
**Permutation tests of peptide embeddings in LncPNdeep.** For each embedding type (Average, Fake, Max peptide), we randomly permuted the embeddings 300 times in the human test dataset and recorded model performance. Violin plots show the distribution of (A) accuracy, (B) sensitivity, and (C) specificity across the permutations. The dashed horizontal lines indicate the mean performance.

To evaluate the contribution of peptide embeddings to prediction accuracy, we performed permutation experiments in which each type of peptide embedding was independently randomized within the human test dataset. For each permutation, we assessed the performance of the LncPNdeep model, repeating the procedure 300 times to ensure robust statistical analysis. The resulting violin plots display the distribution of performance metrics across permutations (Fig. 2).

Among the three embedding types, permuting the Average Peptide Embedding resulted in the largest reduction in mean accuracy and specificity, indicating that ORF-derived peptide representations provide the most informative signal for classification (Table .5). In contrast, permuting the Fake Peptide Embedding produced the smallest decreasing. However, it still led to a consistent decrease in specificity. This suggests that even weaker peptide representations contribute complementary information when integrated through the fused deep learning architecture, rather than being redundant features.

**Table 5:** Performance comparison of LncPNdeep with permutated peptide embeddings. P-values were derived using two-tailed t-tests. The results indicate that removing specific embeddings from the full model leads to a significant degradation in performance. Performance metrics are reported as mean (SD).

	Accuracy	Specificity	Sensitivity
- Average Peptide	0.902(0.002) *p* < 0.001***	0.808(0.004) *p* < 0.001***	0.948(0.001) *p* < 0.001***
- Fake Peptide	0.937(0.001) *p* < 0.001***	0.914(0.002) *p* < 0.001***	0.949(0.001) *p* < 0.001***
- Max Peptide	0.928(0.001) *p* < 0.001***	0.890(0.03) *p* < 0.001***	0.947(0.001) *p* < 0.001***

Above all, these results demonstrate that the performance gains of LncPNdeep arise from joint representation learning across multiple peptide and nucleotide embeddings. The inclusion of multiple peptide-level representations enhances model robustness and discrimination ability.

### 3.4 Comparative analysis of peptide embedding representations

To better understand the role of different peptide embedding strategies, we conducted a comparative analysis of their feature space distributions. This analysis is designed to answer two key questions: (1) Do the three embedding strategies occupy similar or distinct regions in feature space?(2) Does the Fake peptide embedding behave as an artificial artifact, or does it align with biologically derived embeddings? In the analysis, we applied Principal Component Analysis (PCA) to jointly analyze the three embedding types in a shared low-dimensional space. Specifically, a subset of samples from each embedding type was concatenated into a single matrix. We visualize pairwise relationships between the first four principal components (PC1–PC4). Each point in the scatter plot corresponds to one transcript, colored by embedding type.

**Fig. 3:**
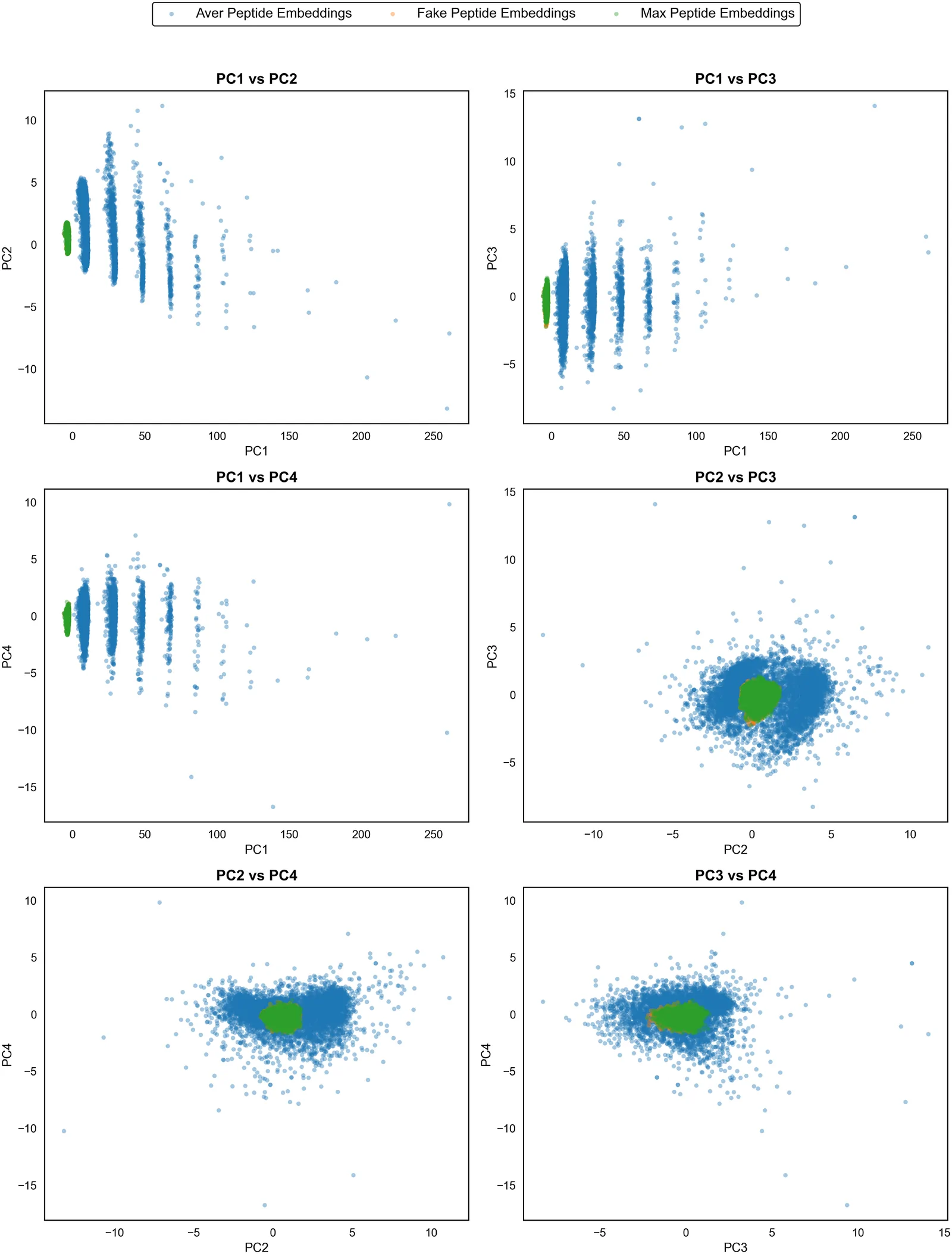
**Principal Component Analysis of Aver, Fake, and Max Peptide Embeddings.** The result reveals strong overlap between Max and Fake Representations. Indicating that Fake peptide embedding aligns closely with the distribution of peptide-derived embeddings (Max).

In Fig. 3, Average peptide embeddings exhibit substantially higher variance and dominate the principal components, indicating that they capture the majority of sequence-dependent information. In contrast, Max and Fake peptide embeddings are concentrated in a low-variance region of the feature space and show strong overlap, suggesting that they encode similar information. Additionally, we evaluated various embedding strategies to determine the performance for lncRNA/coding RNA classification. Using KNN as the model, we found that Bigbird_256 yielded the highest k-NN accuracy (0.89) compared to other embeddings (Table .6).

**Table 6:** k-NN Accuracy results for nucleotide-transformer and peptide-derived embeddings. The accuracy shows the power of lncRNA and coding RNA binary classification.

Embeddings	KNN Accuracy
Bigbird256	0.862960
Bigbird768	0.894561
Longformer256	0.807961
Max Peptide	0.783045
Aver Peptide	0.787906
Fake Peptide	0.778791

### 3.5 t-SNE clusters of each embedding

To assess the discriminative power of individual and combined embeddings, we performed t-SNE clustering on each embedding type to visualize the separation between coding RNAs and lncRNAs. The results showed noticeable overlap between coding and non-coding RNA clusters for all individual embeddings. In particular, BigBird768 and Longformer256 embeddings produced mixed clusters, indicating that nucleotide embeddings alone are insufficient for accurate classification.

**Fig. 4:**
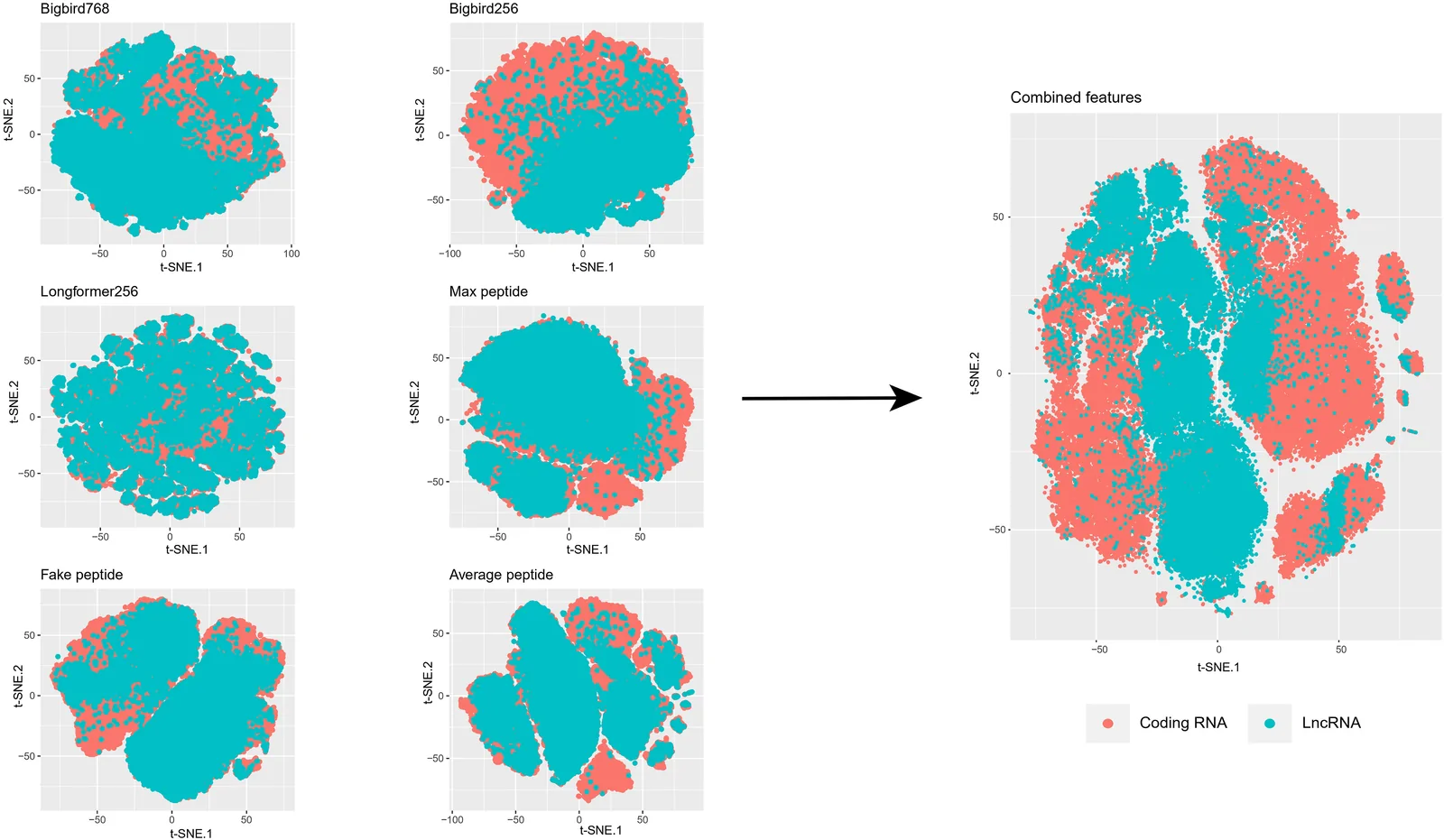
**t-SNE visualization of different embeddings**. Each panel shows 2D t-SNE clustering of lncRNAs (red) and coding RNAs (blue) based on a single embedding: BigBird768, BigBird256, Longformer256, Max peptide, Fake peptide, or Average peptide. Substantial overlap between classes is observed when using individual embeddings. The rightmost panel displays clustering using the concatenated feature space combining all six embeddings, where lncRNAs and coding RNAs exhibit much clearer separation. This highlights the benefit of integrating nucleotide and peptide information for classification.

Peptide embeddings and BigBird256 displayed slightly improved separation; however, substantial overlap between lncRNA and coding RNA clusters persisted (Fig. 4). These findings suggest that relying exclusively on either nucleotide or peptide embedding does not yield optimal classification.

Conversely, t-SNE visualization using the combination of all six embeddings revealed much clearer separation between coding RNAs and lncRNAs, confirming that integrating multiple types of embeddings markedly enhances classification performance.

### 3.6 Computational resources

Pretraining-stage compute. Pretraining was run on a single NVIDIA V100 GPU (16 GB) with approximately 50 GB of CPU memory and required roughly 5 days of wall-clock time on this setup. Wall-clock runtime can vary with hardware (GPU/CPU, RAM, storage throughput) and software configuration; we report the above as the reference runtime for the pretraining stage.

Downstream-stage compute. All language models used in LncPNdeep (BigBird, Longformer, ProtTrans) are pretrained and applied only for embedding extraction, without fine-tuning. Embedding extraction and model training were conducted on a single GPU (RTX 4080 Super) and are feasible in cloud-based notebook environments such as Google Colab. For one transcript prediction, the major time consuming comes from embedding extraction, which needs 1.5s per transcript. Once embeddings are generated, the downstream CNN–BiLSTM classifier requires limited memory and computational resources.

## 4 Discussion

Accurate classification of lncRNAs is essential for advancing our understanding of transcript function, regulation, and their roles in human health and disease. Most existing computational tools rely predominantly on nucleotide sequence features, often neglecting the potential significance of peptide information encoded by some RNAs [[9], [10], [11], [12], [13], [14], [15]]. To address this gap, we developed LncPNdeep, a novel deep learning framework integrating nucleotide and peptide embeddings derived from large language models, enabling a richer, multi-modal characterization of transcripts.

Our benchmarking demonstrates that LncPNdeep achieves state-of-the-art performance on the human RNA dataset, outperforming traditional machine learning algorithms [[9], [10], [11], [12]] and established lncRNA identification methods across multiple metrics, including accuracy, specificity, and sensitivity. Notably, LncPNdeep maintained robust performance across diverse vertebrate and fungal species [36,37], indicating generalizability and reliability in non-model organisms. This cross-species capability is particularly relevant for comparative transcriptomics and could facilitate research in evolutionary biology, environmental genomics, and the investigation of lncRNAs in emerging model systems. While LncPNdeep shows promising cross-species results, notably in vertebrates, performance declines in fungal species likely reflect differences in biological context, particularly with respect to ORF structure and functional characterization [43,44].

Permutation analysis shows that peptide-level information substantially improves predictive accuracy. Recent work indicates that a subset of lncRNAs produce functional peptides, as detected by ribosome profiling and mass spectrometry [22,23]. However, for RNAs lacking canonical ORFs, the use of “fake peptide embeddings” is a computational device designed for input consistency rather than a representation of biological reality. Our results show these embeddings contribute primarily to model regularization, with only marginal improvements in predictive accuracy. We stress that such features do not imply biological translation or function regarding coding potential remain theoretical until validated in future wet-lab investigations.

Despite robust performance, several limitations remain. The biological relevance of peptide embeddings, especially for “fake” peptides, requires experimental validation. Reliance on curated annotations introduces bias, as transcript labels often encode ORF features, confounding coding/non-coding distinctions. Benchmarking is limited to well-annotated species; accuracy may be lower for ambiguous transcripts, pseudogenes, and under-annotated species. Although cross-validation and regularization strategies reduce overfitting, further evaluation on independent datasets is needed to fully establish generalizability.

Additionally, the architectural flexibility of LncPNdeep allows for the inclusion of diverse data types. Incorporating Ribo-seq information as an additional modality—supported by its own feature embedding represents a promising avenue for improving model performance and biological relevance.

Taken together, our findings position LncPNdeep as a versatile tool for lncRNA classification, harnessing the power of large language models to bridge critical gaps in non-coding transcriptomics. By providing both computational accuracy and biologically relevant hypotheses, this work sets the stage for deeper investigation of lncRNA function, regulation, and potential biological roles. Future work will focus on benchmarking LncPNdeep against more challenging and ambiguous transcript categories, including pseudogenes and borderline coding–non-coding transcripts, as well as integrating experimentally validated ORF data to refine peptide-level predictions.

## 5 Conclusion

In summary, LncPNdeep represents a significant advancement in the computational classification of lncRNAs by integrating both nucleotide and peptide-level sequence features using state-of-the-art language models. Our model consistently outperforms traditional machine learning approaches and established lncRNA detection tools, providing robust accuracy across diverse species. Importantly, the inclusion of peptide embeddings enhances predictive performance while providing new insights into the functional landscape of lncRNAs.

LncPNdeep serves as a resource for hypothesis generation and candidate prioritization in transcriptome annotation, supporting informed experimental follow-up rather than substituting for it. Future work will focus on incorporating additional sequence and structural features, expanding coverage to other non-coding RNA types, and experimentally validating predicted lncRNA-derived peptides. Collectively, our work demonstrates the potential of GenAI models in computational transcriptomics and emphasizes the ongoing need for integration with empirical data.

LncPNdeep achieves the highest accuracy (97.1%), specificity (96.7%), and sensitivity (98.0%) to identify lncRNAs, outperforming all baselines.

## CRediT authorship contribution statement

**Zongrui Dai:** Conceptualization, Data curation, Formal analysis, Methodology, Software, Visualization, Writing – original draft, Writing – review & editing. **Feiyang Deng:** Data curation, Formal analysis, Methodology, Software, Visualization, Writing – original draft, Writing – review & editing. **Hsiao H. Sung:** Writing – original draft, Writing – review & editing.

## Declaration of competing interest

The authors declare that they have no known competing financial interests or personal relationships that could have appeared to influence the work reported in this paper.
